# Carbamylation of elastic fibers is a molecular substratum of aortic stiffness

**DOI:** 10.1038/s41598-021-97293-5

**Published:** 2021-09-08

**Authors:** Manon Doué, Anaïs Okwieka, Alexandre Berquand, Laëtitia Gorisse, Pascal Maurice, Frédéric Velard, Christine Terryn, Michaël Molinari, Laurent Duca, Christine Piétrement, Philippe Gillery, Stéphane Jaisson

**Affiliations:** 1 Laboratoire de Biochimie Médicale et Biologie Moléculaire, CNRS/URCA UMR N° 7369 MEDyC Matrice Extracellulaire et Dynamique Cellulaire, Team 2 “Matrix Aging and Vascular Remodeling”, Faculté de Médecine, University of Reims Champagne-Ardenne, 51 Rue Cognacq-Jay, 51095 Reims, France; 2 LRN EA 4682 Laboratoire de Recherche en Nanosciences and NanoMat’ Platform, University of Reims Champagne-Ardenne, Reims, France; 3 BIOS EA 4691 Biomatériaux et Inflammation en site osseux, University of Reims Champagne-Ardenne, Reims, France; 4 PICT Platform, University of Reims Champagne-Ardenne, Reims, France; 5 IPB, CNRS UMR N°5248 CBMN Institute of Chemistry and Biology of Membranes and Nanoobjects, University of Bordeaux, Bordeaux, France; 6 Department of Pediatrics (Nephrology Unit), University Hospital of Reims, Reims, France; 7 Department of Biochemistry-Pharmacology-Toxicology, University Hospital of Reims, Reims, France

**Keywords:** Cardiovascular biology, Chemical modification, Atomic force microscopy, Vascular diseases

## Abstract

Because of their long lifespan, matrix proteins of the vascular wall, such as elastin, are subjected to molecular aging characterized by non-enzymatic post-translational modifications, like carbamylation which results from the binding of cyanate (mainly derived from the dissociation of urea) to protein amino groups. While several studies have demonstrated a relationship between increased plasma concentrations of carbamylated proteins and the development of cardiovascular diseases, molecular mechanisms explaining the involvement of protein carbamylation in these pathological contexts remain to be fully elucidated. The aim of this work was to determine whether vascular elastic fibers could be carbamylated, and if so, what impact this phenomenon would have on the mechanical properties of the vascular wall. Our experiments showed that vascular elastin was carbamylated in vivo. Fiber morphology was unchanged after in vitro carbamylation, as well as its sensitivity to elastase degradation. In mice fed with cyanate-supplemented water in order to increase protein carbamylation within the aortic wall, an increased stiffness in elastic fibers was evidenced by atomic force microscopy, whereas no fragmentation of elastic fiber was observed. In addition, this increased stiffness was also associated with an increase in aortic pulse wave velocity in ApoE^−/−^ mice. These results provide evidence for the carbamylation of elastic fibers which results in an increase in their stiffness at the molecular level. These alterations of vessel wall mechanical properties may contribute to aortic stiffness, suggesting a new role for carbamylation in cardiovascular diseases.

## Introduction

Cardiovascular diseases usually result from a combination of various events including alterations of vascular wall composition. Such modifications, which appear during aging and are enhanced in chronic diseases like diabetes mellitus or chronic renal failure, are responsible for decreased compliance and resilience of vessels, associated with an increase in their stiffness. Arterial stiffness is considered to be an independent predictor of cardiovascular events^1–3^ and is mostly explained by an important structural remodeling occurring within the three layers of the vascular wall, especially in the media^1^.

The tunica media of the arterial wall is composed of lamellar units defined by fenestrated sheets of elastic fibers, separated by a region containing vascular smooth muscle cells, thinner elastic fibers, collagens (mostly types I and III) and proteoglycans^4^. Collagens and elastin are the most abundant extracellular matrix (ECM) proteins in the vascular wall and play a crucial role for ensuring its mechanical and physical properties. Indeed, elastic fibers are responsible for media vascular elasticity and recoil whereas collagen fibers confer resistance properties to the vessels^5^. Thus, any modification in vascular ECM composition has a profound impact on their mechanical properties.

ECM proteins like collagens and elastin are long-lived proteins exposed to molecular aging consisting of a combination of irreversible modifications, including degradation processes and chemical reactions, responsible for alterations of protein structural and functional properties. For instance, elastic fibers are subjected to a progressive proteolysis which occurs during aging or under pathological conditions^2^. Another phenomenon which participates in molecular aging of ECM proteins is represented by the so-called "non-enzymatic post-translational modifications" (NEPTMs)^6^. These reactions correspond to the spontaneous binding of small metabolites to functional groups of proteins. A typical NEPTM is glycation, which is characterized by the reaction between sugars (or their metabolites) and the amino groups of proteins, followed by molecular rearrangements resulting in the formation of advanced glycation end-products (AGEs). Several studies have already provided evidence for the occurrence of glycation of arterial elastic fibers in various species, including humans^7–9^, rats^10^, pigs^11^ or mice^12,13^, and suggested its role in arterial stiffening^10,11,14^.

**Figure 1:**
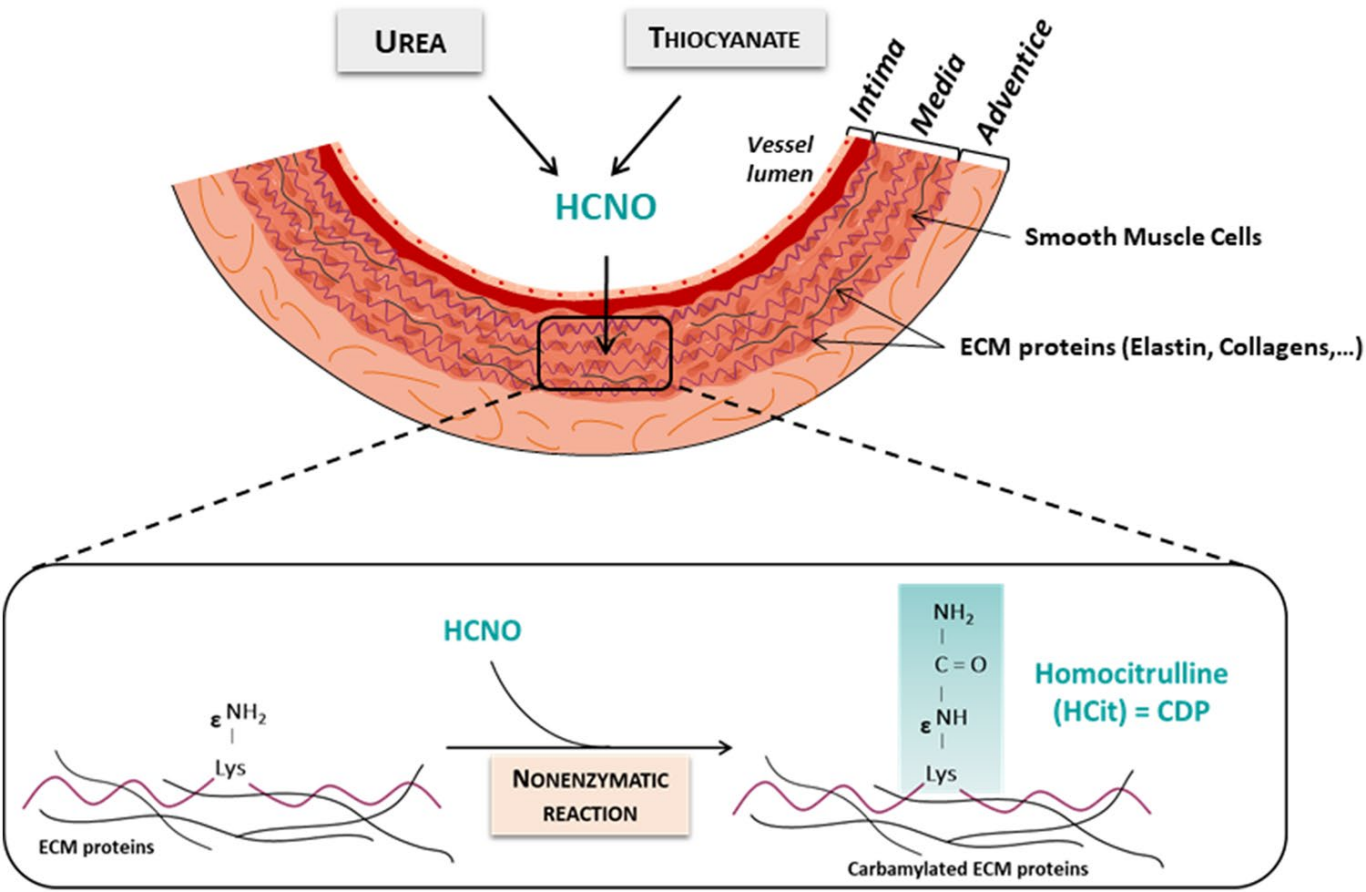
Schematic representation of extracellular matrix protein carbamylation within the arterial wall. *CDP* carbamylation-derived product, *ECM* extracellular matrix.

A more recently described NEPTM is carbamylation which is triggered by the reactive metabolite cyanate^15^. Cyanate mainly derives from the spontaneous dissociation of urea and its reactive form, isocyanic acid, binds to the amino groups of proteins. Its reaction with the ε-amino groups of lysine side chains leads to the formation of homocitrulline (HCit), the most characteristic carbamylation derived-product (CDP) (Fig. 1). The relation between protein carbamylation and cardiovascular diseases has already been demonstrated. For instance, plasma concentrations of CDPs are associated with the occurrence of cardiovascular events and mortality in both uremic and non-uremic patients^16–18^. At the molecular level, several studies have clearly shown the deleterious role of carbamylated lipoproteins in various steps of the atherosclerotic process^19–21^. However, there are no data in the literature reporting the occurrence and consequences of carbamylation of vascular elastic fiber. Our team has already shown that elastic fibers in skin were carbamylated during aging^22^, suggesting that vascular elastic fibers could also be targets of this reaction. The aim of the present study was to determine if vascular elastic fibers were carbamylated and if carbamylation altered mechanical properties of the vascular wall, with a focus on elastic fibers stiffness, assessed at the molecular level by atomic force microscopy (AFM).

## Results

### Bovine aortic elastin is carbamylated in vivo

**Figure 2:**
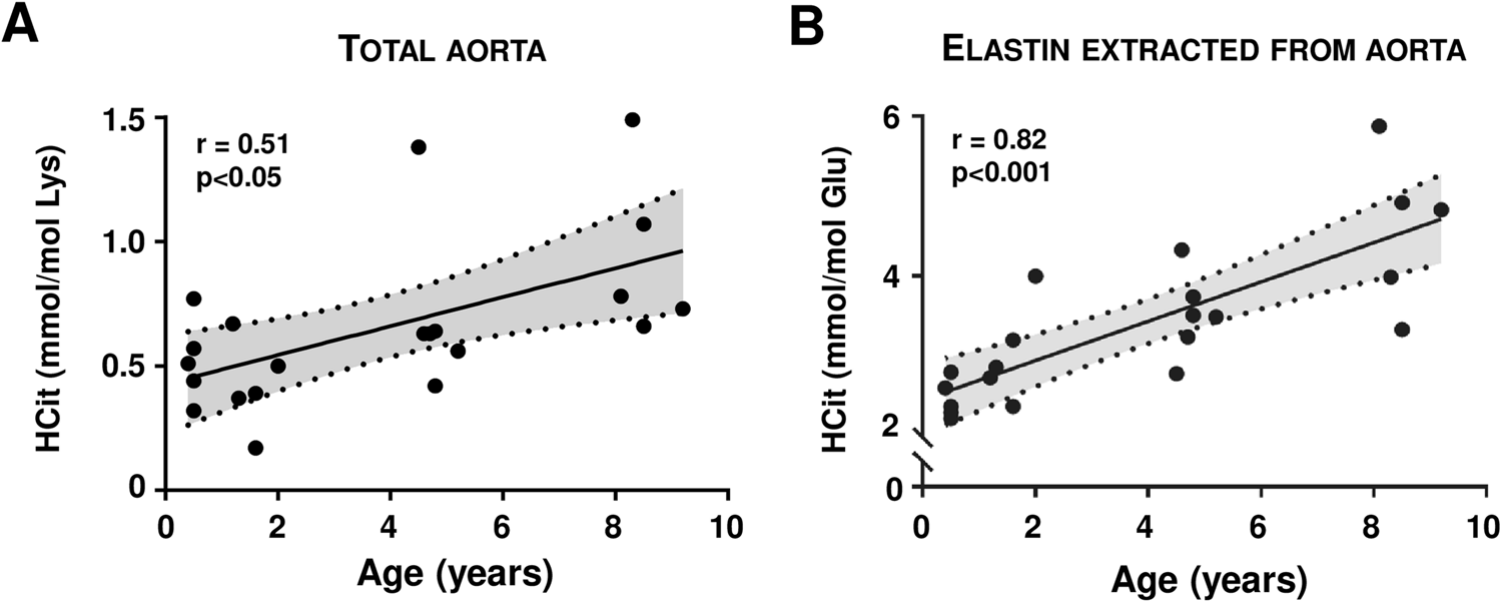
Evolution of carbamylation in bovine aorta with age. Homocitrulline (HCit) was quantified by LC–MS/MS in (**A**) total aorta extracts and in (**B**) aorta elastin of 21 bovine samples. Nonlinear regressions (95% confidence interval; shaded zones) and linear regressions (solid lines) of HCit concentrations over time were calculated. Spearman coefficients of correlation (r) showed a significant HCit increase with age (*p* < 0.05 for total extracts and *p* < 0.001 for elastin).

HCit was first quantified in total extracts of bovine (*Bos taurus*) aortas in order to determine the carbamylation rate of aorta proteins and its evolution with age. Mean HCit concentrations were about 0.46 mmol/mol Lys in 6-month-old bovines and progressively increased with age, reaching 0.92 mmol/mol Lys in 9-year-old animals (Fig. 2A). Quantification of HCit in elastin extracted from the same samples showed that it was carbamylated despite its low number of free lysine residues. HCit concentrations were expressed as ratios to glutamate content in order to avoid any artificial overestimation due to the low lysine content, and ranged from 2.58 mmol/mol Glu in younger bovines to 4.70 mmol/mol Glu in the older animals. Calculation of Spearman’s correlation coefficient showed a significant (*p* < 0.001) increase in elastin carbamylation rate with age (Fig. 2B).

### Neither morphology of bovine elastic fibers nor their sensitivity to elastase are modified by in vitro carbamylation

**Figure 3:**
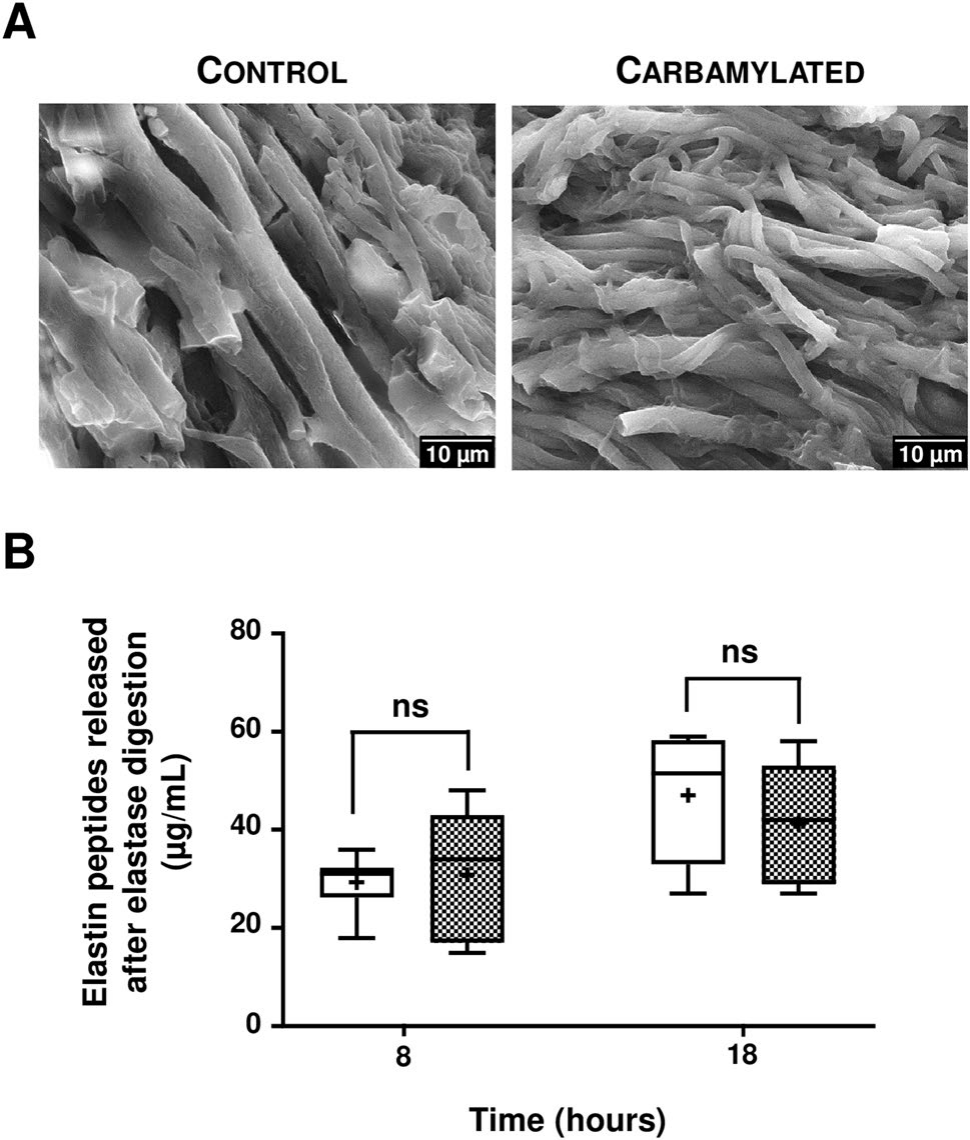
Impact of carbamylation on elastin morphology and sensitivity to elastase. (**A**) Representative SEM images of elastin extracted from bovine aorta and incubated with 100 mM NaCl (Control) or 100 mM NaCNO (Carbamylated) for 24 h at 37 °C. (**B**) Control (open boxes) and carbamylated (grey boxes) elastin samples were incubated with pancreatic elastase (0.1 UI/mL) for 8 and 18 h. Elastin peptides released into the supernatant after precipitation were quantified as described in “Methods” section. Results were expressed as μg/mL and presented as boxplots, in which the error bars represent minimum and maximum values, the horizontal bars and the crosses indicate median and mean values, respectively, and the extremities of the boxes indicate interquartile ranges. Values were compared using the non-parametric Mann–Whitney's U test (*ns* non significant).

Elastin extracted from bovine aorta was carbamylated in vitro by incubation with 100 mM NaCNO (or 100 mM NaCl in control conditions) for 24 h at 37 °C and then observed by scanning electron microscopy (SEM). SEM pictures showed elastin forming a network of intertwined fibers, without any morphological change after carbamylation (Fig. 3A). Sensitivity of carbamylated elastin to proteolysis was then studied by quantifying elastin peptides released after 8 and 18 h of incubation with pancreatic elastase (Fig. 3B). The concentrations of peptides released from carbamylated elastin were not significantly different in comparison with control elastin at both incubation times (8 h-incubation: 29.3 ± 6.0 μg/mL vs 30.8 ± 13.7 μg/mL, 18 h-incubation: 47.0 ± 13.1 μg/mL vs 41.2 ± 12.6 μg/mL), suggesting that carbamylation does not alter sensitivity of aortic elastin to elastase-driven proteolysis.

### Enhancement of carbamylation in NaCNO-fed wild-type mice does not result in elastic fiber degradation

**Figure 4:**
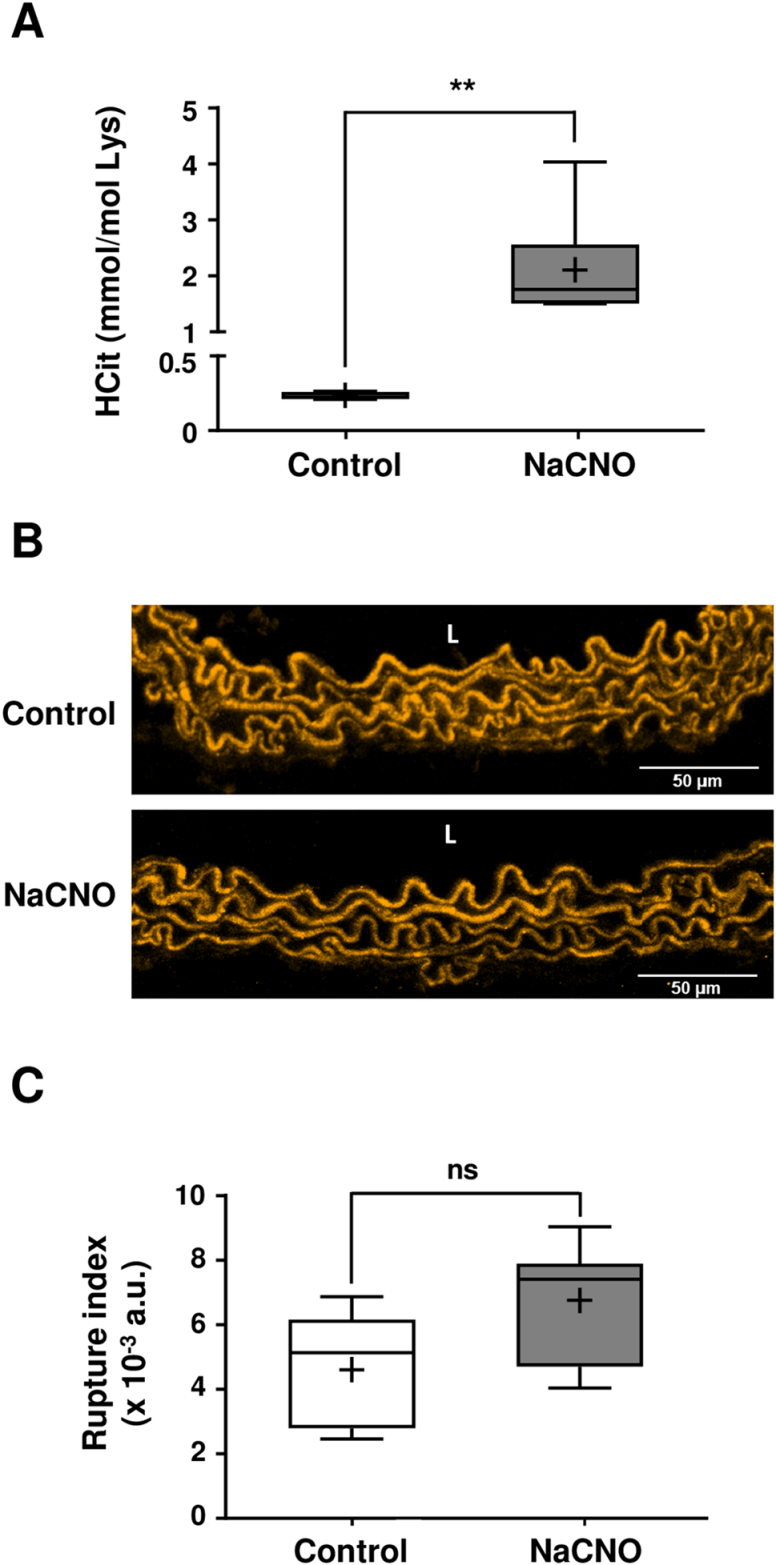
Properties of aortic elastic fibers after carbamylation enhancement in mice. (**A**) HCit was quantified by LC–MS/MS in total aorta extracts in WT mice drinking water supplemented with 1 mM NaCl (control) or 1 mM NaCNO (NaCNO) for 3 weeks. Results were expressed in mmol HCit per mol Lys. (**B**) Representative confocal images of aortic elastic fibers (using autofluorescence properties of elastin). *L* vessel lumen. (**C**) Elastic fiber degradation rates were expressed as rupture index corresponding to the ratio of the number of breaking points to the total area of elastic fibers. For (**A**) and (**C**), results were presented as boxplots, in which the error bars represent minimum and maximum values, the horizontal bars and the crosses indicate median and mean values, respectively, and the extremities of the boxes indicate interquartile ranges. Values were compared using the non-parametric Mann–Whitney's U test (*ns* not significant, **p < 0.01). *a.u.* arbitrary units.

Wild-type (WT) mice received water supplemented with 1 mM NaCNO (or 1 mM NaCl in control conditions) for 3 weeks in order to enhance the carbamylation reaction. HCit concentrations measured in total aorta extracts revealed a significant ninefold increase (*p* < 0.01) of carbamylation rate of proteins in NaCNO-fed mice (2.11 ± 0.97 mmol/mol Lys vs 0.24 ± 0.02 mmol/mol Lys in control group) (Fig. 4A).

Confocal microscopy analysis of aorta cross-sections did not show any morphological differences of aortic elastic fibers in NaCNO-fed mice in comparison with control mice (Fig. 4B). The evaluation of elastic fiber degradation rate based on the calculation of a rupture index (ratio of the number of breaking points to the total area of fibers) did not reveal any significant difference between the two mice groups after 3 weeks of NaCNO diet (Fig. 4C).

### Carbamylation of murine elastic fibers results in an increase in their stiffness

**Figure 5:**
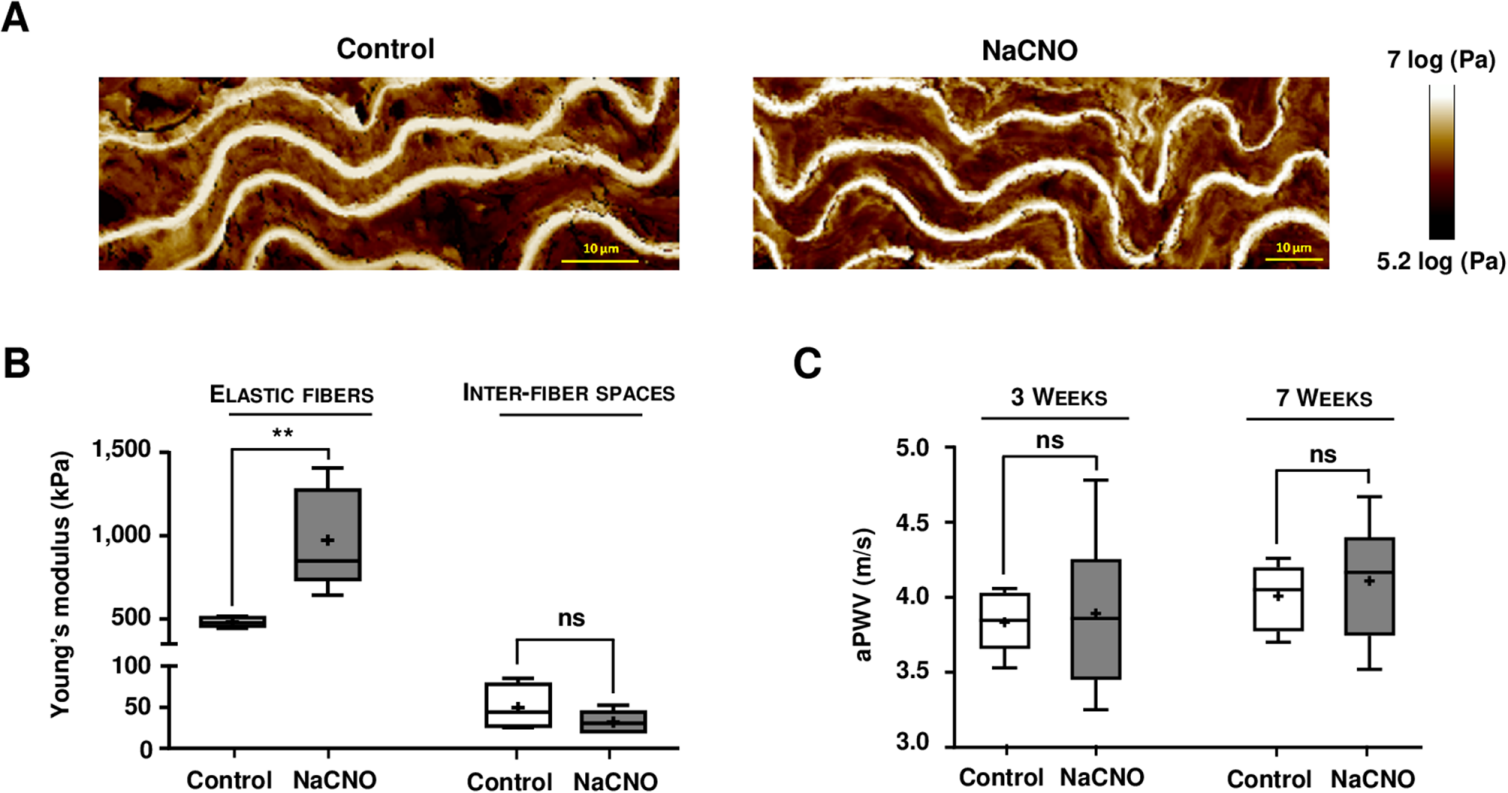
Effect of diet-induced carbamylation on aortic elastic fiber stiffness and aortic pulse wave velocity in WT mice. (**A**) Representative atomic force microscopy pictures showing the Young's modulus (YM) were acquired in 10 μm cross-sections of WT mice aortas using a Peak Force QNM mode. Mice were fed for 3 weeks with water supplemented with 1 mM NaCl (control) or 1 mM NaCNO. (**B**) Quantification of the YM [expressed in kilopascals (kPa)] was carried out directly on elastic fibers and on the inter-fiber spaces. (**C**) Aortic pulse wave was measured at the thoracic and abdominal levels by two Doppler probes in WT mice. Results of aortic pulse wave velocity (aPWV) were expressed in meters per second (m/s). For (**B**) and (**C**), results were presented as boxplots, in which the error bars represent minimum and maximum values, the horizontal bars and the crosses indicate median and mean values, respectively, and the extremities of the boxes indicate interquartile ranges. Values were compared using the non-parametric Mann–Whitney's U test (*ns* not significant, **p < 0.01).

Molecular stiffness of elastic fibers can be assessed by measuring the Young’s modulus (YM), which is calculated after analysis of aortic cross-sections by AFM. In AFM pictures, the modulus is inversely correlated to the color intensity: thus, elastic fibers appear in white, which means that they are stiffer than the brown, inter-fiber spaces (Fig. 5A). After 3 weeks of feeding WT mice with 1 mM NaCNO, the YM of the elastic fibers was increased by + 102% (975 ± 240 kPa vs 482 ± 21 kPa, *p* < 0.01) when compared with controls (Fig. 5B). The stiffness of the inter-fiber spaces, which contains smooth muscle cells and other matrix proteins including collagens, was generally lower, but not significantly so, after carbamylation (32 ± 10 kPa for NaCNO-fed mice *vs* 50 ± 21 kPa for control mice).

In order to determine if the increase in stiffness observed at the molecular level had an impact on the stiffness of the whole vessel, aortic pulse wave velocity (aPWV) measurements were realized after 3 and 7 weeks of NaCNO administration (Fig. 5C). Similar aPWV values were found in the two groups at both analysis times (3 weeks: 3.8 ± 0.2 m/s for control mice vs 3.9 ± 0.5 m/s for NaCNO-fed mice; 7 weeks: 4.0 ± 0.2 m/s for control mice vs 4.1 ± 0.4 m/s for NaCNO-fed mice), suggesting that the increase in elastic fiber stiffness induced by carbamylation had no immediate impact on arterial stiffness. To complement these data, measurement of arterial pressure was performed in both groups and again did not reveal any significant difference (mean arterial pressure at 3 weeks: 81.2 ± 5.4 mmHg for control mice vs 83.2 ± 14.5 mmHg for NaCNO-fed mice; at 7 weeks: 80.3 ± 13.5 mmHg for control mice vs 85.4 ± 4.8 mmHg for NaCNO-fed mice).

**Figure 6:**
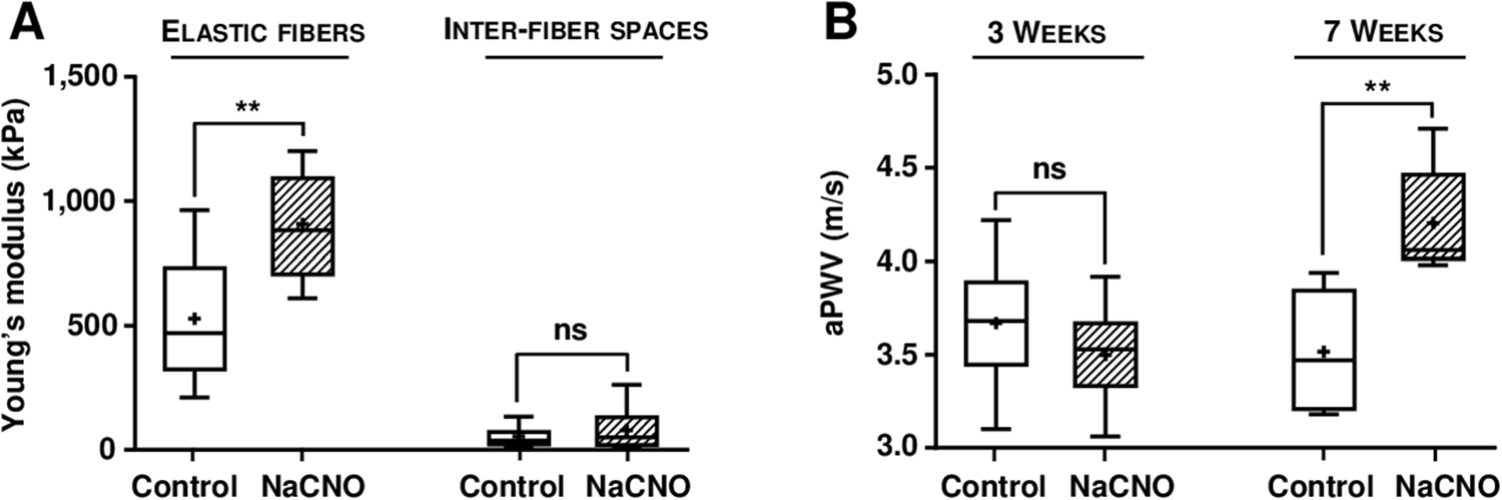
Effect of diet-induced carbamylation on aortic elastic fiber stiffness and aortic pulse wave velocity in ApoE^−/−^ mice. (**A**) Young's modulus (YM) was measured by atomic force microscopy pictures using a Peak Force QNM mode in 10 μm cross-sections of aortas from ApoE^−/−^ mice fed for 3 weeks with water supplemented with 1 mM NaCl (control) or 1 mM NaCNO. Quantification of the YM [expressed in kilopascals (kPa)] was carried out directly on elastic fibers and on the interfiber spaces. (**B**) Aortic pulse wave was measured at the thoracic and abdominal levels by two Doppler probes in ApoE^−/−^ mice. Results of aortic pulse wave velocity (aPWV) were expressed in meters per second (m/s). For (**A**) and (**B**), results were presented as boxplots, in which the error bars represent minimum and maximum values, the horizontal bars and the crosses indicate median and mean values, respectively, and the extremities of the boxes indicate interquartile ranges. Values were compared using the non-parametric Mann–Whitney's U test (*ns* not significant, **p < 0.01).

A last set of experiments was undergone in order to determine if the carbamylation-induced increase in elastic fiber stiffness was also observed in ApoE^−/−^ mice, which are considered a relevant murine model of accelerated vascular aging^23^. ApoE^−/−^ mice were subjected to the same diet as described above (1 mM NaCNO or 1 mM NaCl in drinking water for 3 weeks), and aorta cross-sections were analyzed by AFM. Similar results to those of WT mice were obtained, i.e. an increase in the YM of the elastic fibers under carbamylating conditions. Values increased from 527 ± 256 kPa in the control group to 908 ± 210 kPa in the NaCNO-fed group (+ 72%, *p* < 0.01), whereas no significant difference was observed in YM values measured in the inter-fiber spaces (Fig. 6A).

In contrast with WT mice, whereas no difference of aPWV values was found after 3 weeks of NaCNO diet, a significant increase was noticed (+ 20%, *p* < 0.01) after 7 weeks (control group: 3.5 ± 0.3 m/s vs NaCNO-fed group: 4.2 ± 0.3 m/s), suggesting that carbamylation of vascular wall proteins is associated with a progressive increase in arterial stiffness (Fig. 6B).

## Discussion

Elastic fibers play a key role in the mechanical properties of vessels. However, as there is virtually no renewal of elastin over the lifetime of adult humans, it may be hypothesized that their molecular aging has a major impact on vessel function and could favor many aspects of cardiovascular diseases. Among the phenomena thought to be responsible for molecular aging, enzymatic proteolysis and non-enzymatic post-translational modifications (NEPTMs) such as glycation and carbamylation are the most important. While there is some evidence for the glycation of elastic fibers in human arteries and its subsequent role in arterial stiffening^7,11^, ours is the first study, to our knowledge, to address the effect of carbamylation. The aims of the present study were thus to determine if vascular elastic fibers were prone to carbamylation, and if so, what the consequences on their properties would be.

To date, the relationship between carbamylation and cardiovascular diseases has been evidenced mainly from basic studies suggesting the deleterious role of carbamylated lipoproteins and from clinical trials using carbamylated proteins as biomarkers in these contexts^17,18,24^. By contrast, very few studies have focused on vascular extracellular matrix (ECM) proteins which, because of their long half-lives, are expected to be particularly susceptible to carbamylation. While we have demonstrated that type I collagen was carbamylated at several specific sites which altered its conformation and functions^25^, the case of elastin is more tricky, the number of free lysine residues being very low since most are involved in cross-links like desmosine and isodesmosine. It is estimated that elastin lysine residues represent only about 4% of total amino acids, 16% of them being accessible to modifications^26^. Despite this, our results show that bovine vascular elastin is indeed carbamylated in vivo, and that this rate is of the same magnitude as that of skin elastin^22^.

After having shown that bovine vascular elastin was carbamylated in vivo, we then focused on the impact of carbamylation on the properties of the elastic fibers. In a first step, carbamylation-induced changes on fibers’ morphology and resistance were evaluated. No morphological changes were shown by SEM after in vitro carbamylation of bovine elastin, which may be because the experiments were performed on cross-linked molecules. While carbamylation of tropoelastin may interfere with the process of fiber formation, as observed with carbamylated collagen which exhibits a fibrillogenesis defect^25^, elastogenesis is almost nonexistent after puberty in humans and carbamylation of mature and reticulated fibers is of greater importance than its interference with elastic fiber formation. The degradation assay, performed on bovine elastin with pancreatic elastase, did not demonstrate any modification of sensitivity of carbamylated elastin to proteolysis.

A NaCNO-fed murine model was then used to study elastic fibers’ status in vivo in conditions of increased carbamylation, and did not show any alteration of their morphology. However, AFM analysis of aorta wall showed that a 3-week administration of NaCNO to WT mice led to a significant increase in elastin lamellae stiffness, whereas no difference was observed in the inter-fiber spaces which mainly contain collagens and smooth muscle cells. Indeed, AFM permitted a direct assessment of the impact of carbamylation on elastic fibers’ stiffness by YM measurements. This method had already been used for the measurement of stiffness of different zones within atherosclerotic lesions^27^. These data provide evidence, for the first time to our knowledge, that carbamylation of some of the available lysine residues in elastic fibers is associated with a dramatic increase in their stiffness through a molecular mechanism that remains to be defined. Important differences in YM values between elastic lamellar and inter-fiber spaces were also noted, indicating that the elastic fibers were tenfold stiffer than the inter-fiber spaces, which is consistent with previous observations reported for sheep and human aorta^27,28^.

No data on carbamylation of elastic fibers are available in the literature, which precludes comparison of our data with other studies. One study on glycation of elastin reported opposite effects, i.e. a decrease in stiffness by glycation^29^. However, this in vitro study was performed on elastin extracted from arteries and glycated with very high, non-physiological, concentrations of glucose (i.e*.* 5–10 M). The authors also used a different method for measuring stiffness, further complicating comparison. Besides, glycation and carbamylation are two distinct mechanisms which may lead to opposite effects at the molecular level. For example, while we observed no effect of carbamylation on elastin sensitivity to elastase, glycation of alpha-elastin made it more resistant to neutrophil elastase digestion^30^. An important concept to be borne in mind is the possible competition between these two reactions. Indeed, the presence of glycated elastin in human arteries^7,31^, brought face to face with our data showing carbamylation of bovine elastin, suggests it is subjected to several NEPTMs in vivo. Given the limited number of free lysine residues available, it could be expected that these reactions compete for the modification of elastin, as we have recently shown for collagen^32^, but also contribute simultaneously to the modification of elastic fibers’ properties.

To determine the impact of carbamylation under pathological (i.e. pro-atherosclerotic) conditions, these experiments were repeated in an ApoE^−/−^ murine model. The vascular wall of ApoE^−/−^ mice differs from that of WT mice due to the lack of apolipoprotein E gene expression, thereby promoting dyslipidemia, subintimal inflammation and atherosclerosis development. ApoE^−/−^ mice develop atherosclerotic lesions as early as 10 weeks old and constitute an excellent model of accelerated vascular aging^23^. In this study, feeding ApoE^−/−^ mice with NaCNO resulted in an increase in elastic fibers’ stiffness at the molecular level, but also globally at the vessel level as indicated by the significant increase in aPWV after 7 weeks treatment. Unexpectedly, no variation in aPWV was observed in WT mice. One hypothesis to explain this result could be a delay in the development of arterial stiffness in WT compared with ApoE^−/−^ mice, which are more prone to vascular changes because of their genetic background. Indeed, ApoE^−/−^ mice have been described to develop vascular stiffness (demonstrated by the increase in aPWV) more rapidly than WT mice^33,34^. Further studies remain thus to be performed to better determine the consequences of increased elastic fiber stiffness on functional properties of arteries.

Such findings may be of added value in chronic renal failure, a disease that promotes carbamylation due to hyperuremia and whose complications often include cardiovascular events: it has been demonstrated that carbamylated lipoproteins are more atherogenic^21,24^ and that uremic, pro-carbamylating conditions stimulate calcification processes which also contribute to arterial stiffening^35^. Our data offer a mechanistic explanation of how carbamylation of vascular ECM proteins may contribute, with these other mechanisms, to the progression of vascular diseases in uremic patients.

In conclusion, our study has shown, for the first time, that vascular elastic fibers are prone to carbamylation and that this process is associated with an increase in their stiffness at the molecular level. These alterations may have deep consequences on the mechanical properties of the vascular wall, which suggests that carbamylation, together with other NEPTMs, are crucial factors in the etiology of cardiovascular diseases.

## Methods

All the procedures were carried out in accordance with proper guidelines and regulations.

### Tissue samples and animal models

#### Bovine samples

Bovine (*Bos taurus*) aorta samples were provided by the Soredex Slaughterhouse (Rethel, France) in accordance with French government policies (*Services Vétérinaires d’Inspection, Ministère de l’Agriculture*). Animals included in this study (n = 20) were from 6 months to 9 years old.

#### Mice models

Experiments were performed using C57Bl/6J WT or Apolipoprotein E deficient (ApoE^−/−^) mice purchased from Charles River Laboratories (Calco, Lecco, Italy). Animals were fed ad libitum and housed in a room at a constant ambient temperature and with a 12-h light–dark cycle. All procedures involving mice were approved by the institutional animal care committee of the University of Reims Champagne-Ardenne (registration 56) and the veterinary services of the health and the production in accordance with French government policies (APAFIS#4433-2016030911228135). This study was carried out in compliance with the ARRIVE guidelines.

Cyanate-consuming model: 6-week-old mice (WT and ApoE^−/−^) were randomly assigned to two groups (n = 6 each): one group received drinking water supplemented with 1 mM NaCl (i.e. control conditions), and one group received drinking water supplemented with 1 mM NaCNO (NaCNO-fed group). Water was renewed twice a week during the course of the experiments (3 or 7 weeks).

#### Extraction protocols

#### Total tissue extracts

Bovine (100 mg) or mice (15 mg) aortas were homogenized with 800 µL of 0.5 M acetic acid in Lysing Matrix D™ tubes using the FastPrep-24 System (MP Biomedicals, Illkirch-Graffenstaden, France). After 6 cycles of homogenization (40 s, 6 m/s), samples underwent 10% (m/m) pepsin digestion for 24 h at 37 °C. After 3 additional cycles of homogenization under the same conditions, samples were centrifuged at 14,000*g* for 5 min, and supernatants were collected and stored at − 80 °C before HCit quantification by liquid chromatography coupled to tandem mass spectrometry (LC–MS/MS).

#### Elastin extraction

Bovine aorta samples (200 mg) were ground in a ball mill under liquid nitrogen cooling (Retsch Technology, Eragny sur Oise, France), and elastin was extracted as described previously^22^. Briefly, the protocol consisted in different steps combining washing procedures with buffers and solvents (1 M NaCl, pure ethanol, chloroform:methanol (2:1 vol/vol), ether, acetone) and protein cleavages with cyanogen bromide or trypsin. At each step, a volume of 1.5 mL corresponding buffer was added, samples were centrifuged at 14,000*g* for 2 min, and the supernatant was carefully removed.

### In vitro elastin carbamylation and degradation assay

#### In vitro elastin carbamylation

Elastin was carbamylated by incubating 2 g of extracted bovine elastin with 100 mM NaCNO in 150 mM phosphate buffer pH 7.4 for 24 h at + 37 °C under gentle stirring. A control experiment was carried out in parallel with NaCl instead of NaCNO. Samples were then washed several times with phosphate buffer and dried at 37 °C.

#### In vitro degradation assay

Carbamylated (NaCNO) or control elastin (200 µg) was incubated at a concentration of 1 mg/mL in 50 mM Tris buffer pH 7.0 under gentle stirring with 0.1 U/mL porcine pancreatic elastase (Merck Chimie, Fontenay-sous-bois, France) for 8 and 18 h at + 37 °C (n = 6). The reaction was stopped by adding 0.5% (v/v, final concentration) trifluoroacetic acid (Sigma, Machelen, Belgium). Samples were centrifuged at 10,000*g* for 10 min at + 4 °C, and supernatants were stored at − 80 °C until analysis. The quantification of released elastin peptides used the "Elastin Assay-Fastin™" (Biocolor, INTERCHIM, Montluçon, France). For this purpose, elastin fragments contained in the supernatant were precipitated in an "Elastin Precipitating Reagent buffer". After centrifugation at 10,000*g* for 10 min at + 4 °C, the pellet containing elastin fragments was incubated for 1h30 with 1 mL of Fastin Dye Reagent under orbital shaking under darkness. This mixture was centrifuged (10,000*g*, 10 min, + 4 °C) and the pellet was solubilized in 250 μL of Dye Dissociation Reagent buffer. Absorbance was measured at 513 nm, a calibration curve being established in parallel using a standard containing a known concentration of elastin peptides.

### HCit quantification

HCit was quantified in tissue samples using a previously described liquid chromatography coupled to tandem mass spectrometry (LC–MS/MS) method^22,36^. Briefly, samples were subjected to acid hydrolysis with 6 M HCl for 18 h at 110 °C and hydrolysates were twice evaporated to dryness under a nitrogen stream. Dried samples were resuspended in 100 μL 125 mM ammonium formate containing 1 μM d7-citrulline and 65 μM d8-lysine [used as internal standards (ISs)] and filtered using Uptidisc PTFE Filters (4 mm, 0.45 μm; Interchim). Ten-fold diluted hydrolysates were subjected to LC–MS/MS analysis (API4000; ABSciex) to quantify HCit, Lys or Glu. Liquid chromatography was performed using a Kinetex HILIC Column (100 × 4.6 mm, 2.6 μm; Phenomenex) with 5 mM ammonium formate (pH 2.9) as mobile phase A and 100% acetonitrile as mobile phase B. The flow rate was constant at 0.9 mL/min during all separation steps. Details parameters for LC separation and MS/MS detection have been described elsewhere^22,36^. Results were expressed as ratios to lysine content in total aorta extracts and to glutamate content in elastin extracts.

### Scanning electron microscopy

Dehydrated elastin pellets obtained after drying at 37 °C for 24 h were attached to an aluminum slide using of double-sided carbon tape and covered with a conductive layer (Au–Pd, cathodic evaporator JEOL JFC-1100, 1.2 kV, 8 mA, 10 min) to favor the interactions between the sample and the electron beam. Observation was performed using a field emission electron microscope with Schottky tip (JSM-7900F prime, JEOL Europe SAS, Croissy sur Seine, France) operating with a beam of 20 kV primary energy.

### Evaluation of in vivo degradation of murine aortic elastic fibers

Elastin autofluorescence was measured by analyzing 5 µm cross-sections of aortas from WT mice with a two-Photon-Excited Fluorescence (2-PEF-excitation wavelength 860 nm; emission wavelength between 500 and 535 nm) using a scanning confocal microscope LSM 710 NLO coupled with a Titanium-Sapphire Chameleon femtosecond laser (20 × 0.8NA-COHERENT, Lisses, France). Pictures were analyzed using the Skeleton module, and the Fiji software permitted counting of the breaking points in selected fiber areas. The number of breaking points determined in the entire section was averaged and the degradation rate of elastic fibers was expressed as a rupture index, corresponding to the ratio of the number of breaking points to the total area of the 2-PEF signal.

### Measurement of murine elastic fiber stiffness by atomic force microscopy

Cross-sections of aortas from WT or ApoE^−/−^ mice fed or not with 1 mM NaCNO were analyzed by AFM (Bioscope Catalyst, Bruker, Billerica, USA, driven by the Nanoscope Analysis 1.8 software) coupled to a Nikon Eclipse Ti inverted microscope (Nikon, Tokyo, Japan) in order to determine the stiffness of elastic fibers at the molecular level. Frozen 10 µm aorta cross-sections were thawed for 1 h at room temperature before applying a Krebs–Henseleit solution (118 mM NaCl, 4.7 mM KCl, 1.2 mM KH_2_PO_4_, 1.2 mM MgSO_4_, 25 mM NaHCO_3_, 11 mM glucose, 2.5 mM CaCl_2_, 3 mM ethylenediamine-tetracetic acid (EDTA) pH 7.4) to the sample and waiting for equilibration at + 37 °C. The prepared samples were put onto the AFM stage and observed with Bright Field illumination in order to locate the spots of interest. To get a representative set of values, for each cross section, AFM analyses were performed at 3 different locations of the cross section and each experiment was triplicated leading to 9 different areas analyzed per condition. Experiments were performed in the Krebs–Henseleit buffer using the Peak Force Quantitative NanoMechanical (PFQNM) mode with ScanAsyst-air probes (Bruker, Billerica, USA) with a nominal spring constant of 0.4 N/m and a nominal resonant frequency of 70 kHz. For the PFQNM calibration, the standard supplier protocol was applied to get quantitative measurements of the YM. First, the deflection sensitivity was calibrated before use in the buffer by carrying out indentation ramps on a clean and hard sapphire surface. Then, the cantilever spring constant was calculated before and after each experiment following the thermal tuning method. The last step was to calibrate the curvature radius of the tip by using a standard titanium tipcheck sample. This curvature radius was confirmed by performing a test measurement of the YM of a calibrated known sample. A PeakForce frequency of 0.25 kHz was used in order to maximize the contact time between the tip and the sample and the PeakForce amplitude was set to 2 µm. The distance synchronization parameter was manually and constantly adjusted over time so that the turnaway point of each force curve was exactly at the (x,y) maximum position. Images were captured with a resolution of 256 pixels per line. Once the different AFM images acquired, for the YM calculation, the force curves were extracted from chosen areas in the PFQNM images and the conventional Derjaguin–Muller–Toporov (DMT) model was used to fit the linear part of the extension curve as it was identified as the best suited model according to the tip geometry and the properties of the samples. The YM at each point of the elastic fibers or of the inter-fiber spaces was calculated using a value of the Poisson ratio of 0.5 for our samples considered as incompressible. For each condition, at least 5000 force curves were treated to get the statistical values of the YM for the elastic fibers and the inter-fiber spaces. The analyses were performed at 3 different locations in each cross-section, for a total of 9 cross-sections obtained from 3 different mice.

### Evaluation of murine arterial stiffness in vivo

Measurement of aPWV in WT and ApoE^−/−^ mice, fed or not with 1 mM NaCNO, was performed using the Doppler Flow Velocity system (Indus Instruments, Houston, Texas)^37^. Doppler signals were measured at the level of thoracic and abdominal aortas by using focused 10 MHz and 20 MHz Doppler probes, respectively. Doppler signals were recorded and the distance between the two probes measured, allowing aPWV calculation using the following formula: aPWV (m/s) = distance (m)/Δt (s), where “distance” is the distance between the two probes and Δt is the averaged time between two ejection times.

### Measurement of blood pressure

Arterial pressure was measured using a non-invasive blood pressure measurement system, *i.e.* a tail-cuff sphygmomanometer equipped with the photoplethysmogram Visitech BP-2000 (Visitech Systems, Apex, NC). Mice were accustomed to the procedure for 5 consecutive days before measurement and were monitored weekly during the 7 weeks of the study. For blood pressure measurements, the mice were placed in a warm (37 °C) restraint device with an appropriately sized cuff around the tail. In order to minimize stress-induced blood pressure fluctuations in the animals, all measurements were taken after a 5-min adaptation period. All measurements of diastolic and systolic pressures were taken between 9:00 a.m. and 12:00 a.m., and recordings were averaged from at least 5 consecutive readings using BP-2000 analysis software. Mean arterial pressure was calculated using following formula: [(2 × diastolic) + systolic]/3.

### Statistical analysis

Nonlinear regression (second-order polynomial function with a 95% confidence interval) and linear regression were computed using GraphPad Prism 7.0 software. Non-parametric Spearman’s tests were used for the calculation of correlation coefficients. Values were compared using the non-parametric Mann–Whitney’s U test. Differences were considered statistically significant for a *p* value below 0.05. Values mentioned in the text are expressed as means ± standard deviations.

## Abbreviations

AFM: Atomic force microscopy
AGEs: Advanced glycation end-products
ApoE: Apolipoprotein E
aPWV: Aortic pulse wave velocity
ECM: Extracellular matrix
HCit: Homocitrulline
LC–MS/MS: Liquid chromatography coupled to tandem mass spectrometry
NEPTMs: Non-enzymatic post-translational modifications
SEM: Scanning electron microscopy
WT: Wild-type
YM: Young's modulus

## References

[1] Fleenor BS (2013). Large elastic artery stiffness with aging: Novel translational mechanisms and interventions. Aging Dis..

[2] Greenwald SE (2007). Ageing of the conduit arteries. J. Pathol..

[3] Sutton-Tyrrell K (2005). Elevated aortic pulse wave velocity, a marker of arterial stiffness, predicts cardiovascular events in well-functioning older adults. Circulation.

[4] Cocciolone AJ (2018). Elastin, arterial mechanics, and cardiovascular disease. Am. J. Physiol. Heart Circ. Physiol..

[5] Duca L (2016). Matrix ageing and vascular impacts: Focus on elastin fragmentation. Cardiovasc. Res..

[6] Jaisson S, Gillery P (2010). Evaluation of nonenzymatic posttranslational modification-derived products as biomarkers of molecular aging of proteins. Clin. Chem..

[7] Konova E, Baydanoff S, Atanasova M, Velkova A (2004). Age-related changes in the glycation of human aortic elastin. Exp. Gerontol..

[8] Yamamoto Y (2002). Possible involvement of increased glycoxidation and lipid peroxidation of elastin in atherogenesis in haemodialysis patients. Nephrol. Dial. Transplant..

[9] Zarkovic K (2015). Elastin aging and lipid oxidation products in human aorta. Redox. Biol..

[10] Tomizawa H, Yamazaki M, Kunika K, Itakura M, Yamashita K (1993). Association of elastin glycation and calcium deposit in diabetic rat aorta. Diabetes Res. Clin. Pract..

[11] Wang Y, Zeinali-Davarani S, Davis EC, Zhang Y (2015). Effect of glucose on the biomechanical function of arterial elastin. J. Mech. Behav. Biomed. Mater..

[12] Coquand-Gandit M (2017). Chronic treatment with minoxidil induces elastic fiber neosynthesis and functional improvement in the aorta of aged mice. Rejuvenation Res..

[13] Fleenor BS (2012). Sodium nitrite de-stiffening of large elastic arteries with aging: Role of normalization of advanced glycation end-products. Exp. Gerontol..

[14] Sell DR, Monnier VM (2012). Molecular basis of arterial stiffening: Role of glycation—a mini-review. Gerontology.

[15] Jaisson S, Pietrement C, Gillery P (2018). Protein carbamylation: Chemistry, pathophysiological involvement, and biomarkers. Adv. Clin. Chem..

[16] Jaisson S (2015). Increased serum homocitrulline concentrations are associated with the severity of coronary artery disease. Clin. Chem. Lab. Med..

[17] Koeth RA (2013). Protein carbamylation predicts mortality in ESRD. J. Am. Soc. Nephrol..

[18] Wang Z (2007). Protein carbamylation links inflammation, smoking, uremia and atherogenesis. Nat. Med..

[19] Apostolov EO, Ray D, Savenka AV, Shah SV, Basnakian AG (2010). Chronic uremia stimulates LDL carbamylation and atherosclerosis. J. Am. Soc. Nephrol..

[20] Asci G (2008). Carbamylated low-density lipoprotein induces proliferation and increases adhesion molecule expression of human coronary artery smooth muscle cells. Nephrology (Carlton).

[21] Basnakian AG, Shah SV, Ok E, Altunel E, Apostolov EO (2010). Carbamylated LDL. Adv. Clin. Chem..

[22] Gorisse L (2016). Protein carbamylation is a hallmark of aging. Proc. Natl. Acad .Sci. USA.

[23] Plump AS (1992). Severe hypercholesterolemia and atherosclerosis in apolipoprotein E-deficient mice created by homologous recombination in ES cells. Cell.

[24] Apostolov EO (2013). Carbamylated-oxidized LDL: Proatherosclerotic effects on endothelial cells and macrophages. J. Atheroscler. Thromb..

[25] Jaisson S (2006). Impact of carbamylation on type I collagen conformational structure and its ability to activate human polymorphonuclear neutrophils. Chem. Biol..

[26] Han K, Davril M, Moczar M, Moczar E (1981). Accessibility of epsilon-amino groups of lysine to guanidination in kappa-elastin from bovine ligamentum nuchae. Paroi Arterielle.

[27] Rezvani-Sharif A, Tafazzoli-Shadpour M, Avolio A (2019). Mechanical characterization of the lamellar structure of human abdominal aorta in the development of atherosclerosis: An atomic force microscopy study. Cardiovasc. Eng. Technol..

[28] Akhtar R (2016). Frequency-modulated atomic force microscopy localises viscoelastic remodelling in the ageing sheep aorta. J. Mech. Behav. Biomed. Mater..

[29] Stephen EA, Venkatasubramaniam A, Good TA, Topoleski LD (2014). The effect of glycation on arterial microstructure and mechanical response. J. Biomed. Mater. Res. A.

[30] Yoshinaga E (2012). N(epsilon)-(carboxymethyl)lysine modification of elastin alters its biological properties: Implications for the accumulation of abnormal elastic fibers in actinic elastosis. J. Invest. Dermatol..

[31] Sakata N (2003). Modification of elastin by pentosidine is associated with the calcification of aortic media in patients with end-stage renal disease. Nephrol. Dial. Transplant..

[32] Nicolas C (2019). Carbamylation and glycation compete for collagen molecular aging in vivo. Sci. Rep..

[33] Hartley CJ (2000). Hemodynamic changes in apolipoprotein E-knockout mice. Am. J. Physiol. Heart Circ. Physiol..

[34] Wang YX (2000). Increased aortic stiffness assessed by pulse wave velocity in apolipoprotein E-deficient mice. Am. J. Physiol. Heart Circ. Physiol..

[35] Mori D (2018). Protein carbamylation exacerbates vascular calcification. Kidney Int..

[36] Jaisson S (2018). Measurement of homocitrulline, a carbamylation-derived product, in serum and tissues by LC-MS/MS. Curr. Protoc. Protein Sci..

[37] Reddy AK (2009). Multichannel pulsed Doppler signal processing for vascular measurements in mice. Ultrasound Med. Biol..

